# Supplementary material for: Object permanence in domestic cats (Felis catus) using violation-of-expectancy by owner and stranger
Source: PLoS One. 2025 Jul 9;20(7):e0312225. doi: 10.1371/journal.pone.0312225 (PMC12240391; doi:10.1371/journal.pone.0312225)
Supplement: Attachment — Submitted filename: response_to_reviewers_28_02_25.docx [file pone.0312225.s002.docx]

We thank the Editor and all reviewers for providing thoughtful improvements for this manuscript. We have revised the manuscript to fit the journal requirements of *PLOS ONE*. We appreciate the concerns regarding the expectations of the box switching and have further clarified some aspects of the study design that were identified by reviewers. We have carefully considered and responded to each of the comments in turn.

**Journal Requirements**

**Point 1:** Please ensure that your manuscript meets PLOS ONE's style requirements, including those for file naming. The PLOS ONE style templates can be found at

[https://journals.plos.org/plosone/s/file?id=wjVg/PLOSOne_formatting_sample_main_body.pdf](https://url.uk.m.mimecastprotect.com/s/p4lCCp2XETnzE6xwUGsEHGQkTP?domain=journals.plos.org) and

[https://journals.plos.org/plosone/s/file?id=ba62/PLOSOne_formatting_sample_title_authors_affiliations.pdf](https://url.uk.m.mimecastprotect.com/s/UfknCqZMGF8O6w72tNtxHEcZLt?domain=journals.plos.org)

*Response: Apologies for these formatting oversights. We have checked the templates from the above links and have now formatted the manuscript to meet PLOS ONE’s style requirements for file naming.*

**Point 2:** Please match your authorship list in your manuscript file and in the system.

*Response: The authorship list is now formatted to meet PLOS ONE’s style requirements and is matched in the system.*

**Point 3:** Please include a caption for figure 1a and 2.

*Response: The figure captions were included in the original manuscript immediately after the first paragraph in which the figure is cited but have now been formatted for PLOS ONE standards. Because this comment has been similarly noted by Reviewer 3, we are hoping this is satisfactory.*

*I had previously referred to the submission guidance: “Figure captions must be inserted in the text of the manuscript, immediately following the paragraph in which the figure is first cited (read order). Do not include captions as part of the figure files themselves or submit them in a separate document.”*

**Point 4:** Your ethics statement should only appear in the Methods section of your manuscript. If your ethics statement is written in any section besides the Methods, please move it to the Methods section and delete it from any other section. Please ensure that your ethics statement is included in your manuscript, as the ethics statement entered into the online submission form will not be published alongside your manuscript.

*Response: The ethics statement has now been moved to the Methods section of the manuscript.*

*At the request of the PLOS ONE office, we have additionally updated the DOI that links to our data and supplementary materials to reflect the PLOS ONE guidance for online repositories (we previously used Mendeley Data, but we have now moved our data to an approved repository of figshare). Please note the DOI will become active once approved by my institution.*

**Reviewer Comments**

Major points

**Point 5:** I understand that the research question is whether cats can pass Stage 6 of object permanence. If this is the case, it seems that the Event trials implement the Invisible Displacement procedure of Stage 6. From the perspective of the cats, it could be perceived that the boxes were being swapped in both Consistent and Violation events. Therefore, if cats possess the cognitive ability to pass Stage 6, then Violation events may not actually be violations but rather consistent. I found it difficult to evaluate the paper as I did not fully understand the authors’ logic on this point. Since the results differed from the authors' predictions, reinterpreting the results based on the logic I have suggested would amount to HARKing, which should be avoided. However, I would like the authors to clarify this point thoroughly.

*Response: We predicted that cats would show more interest in the toy (longer gaze durations, more physical touches with the toy) when it re-appeared out of the box it was not seen to be originally hidden (violation event) in comparison to when it re-appeared out of the box it was seen to be originally hidden (consistent event). We maintain this prediction in the manuscript based on the findings of previous literature on human infants and domestic dogs and to avoid HARKing. However, contrary to our prediction, we found that cats showed more interest in the toy in consistent events in comparison to violation events.*

*Because of these contradictory findings, the reviewer provided an alternative explanation that the cats might have assumed the boxes were switched in both conditions (consistent and violation events), hence why the cats showed more interest in the consistent events when the boxes were not switched. We lack a ready explanation for why the cats would have made such an assumption, but this is a valid alternative interpretation.*

*Before the event trials, cats were shown a SVD trial performed by either the owner or the researcher. This SVD trial would not have primed the cats to assume a box switch. If a cat did not independently search for the toy in the allotted time, the box manipulator would always reveal the toy coming out of the same box that it was hidden in for SVD trials. Furthermore, the presentation order of either the consistent or violation event was randomised, so it is unlikely that cats would have had expectations about the location of the toy based on previous presentations. Moreover, the cats had longer gaze durations at the toy when presented with the consistent event before the violation event (which does lend evidence to the hypothesis that cats were expecting a box switch). However, because this analysis was grouped across both event types, the relative proportion of gazing at the toy in either the consistent or violation conditions is unclear. For example, a cat may have had longer gaze durations at the toy when presented with the consistent event first, but that cat may have gazed for longer durations during the violation trials, even if they were presented after the consistent trials.*

*As we see it, the fundamental facts in play, here, are (a) that the cats did discriminate between consistent and violation conditions in their gaze durations and toy touching, (b) that the pattern is opposite to that reported for dogs and young children, who also discriminate the conditions, albeit in the opposite direction (tend to engage with the object in the violation conditions), and (c) nobody directly measures object permanence as a cognitive event; rather, there is a longstanding tendency to interpret discriminations in terms of object representations.*

*It is worth noting that if the cats assumed switching, this would constitute evidence for Stage 6 object permanence, at the cost of an objective assay for the capacity—that is, gazing longer at the toy in both the consistent (cats) and violation (humans, dogs) conditions would be taken, variously, as evidence for object permanence, thus invalidating the entire protocol.*

*We did not have any definitive evidence for cats to either have a) expected a switch, or b) expected a non-switch. It is theoretically possible that they could have expected a switch, but this would have far-reaching implications for the whole protocol. We did not want to burden the manuscript with this whole explanation but remain keen to revise further if the editor or reviewer require it. We have no direct way to address this concern raised by the reviewer in the current manuscript.*

*Nonetheless, in the manuscript itself, we have included a brief sentence that addresses this valid point raised by the reviewer.*

*Lines 449 - 453 “As mentioned by a reviewer, it is unlikely, but not impossible, that cats were primed to expect a box switch from the SVD trials (in which cats were always shown the toy re-appearing out of the same box it was hidden) or from the randomised presentation order of the consistent and violation event trials.”*

**Point 6:** In addition to the main factors of whether the manipulator was the owner or the researcher, and whether the event was a Violation or Consistent, the authors have also comprehensively examined factors such as the cat’s age, sex, multi-cat household, outdoor access, and breed. However, when examining numerous factors simultaneously, there is a risk of obtaining significant results by chance. Since no predictions were made for factors beyond the primary factors, these should be considered exploratory analyses, making it difficult to draw definitive conclusions from the results of this experiment.

*Response: We have now clarified that our analyses of lifestyle factors were carried out as exploratory analyses throughout the manuscript. As this reviewer correctly explains, we have now also included the limitation that there is a risk of obtaining significant results by chance by examining numerous factors simultaneously.*

*Lines 120 – 122 “Furthermore, we also carried out exploratory analyses for whether demographic factors (such as cat age, cat sex, living in a multi-cat household, outdoor access, or breed) influenced any observed behaviours during SVD and event trials.”*

*Lines 284 – 287 “For both SVD and event trials, we carried out exploratory analyses for numerous lifestyle factors (cat age, cat sex, multi-cat household, outdoor access, and breed) and their effects on our outcome variables.”*

*Lines 508 – 510 “Furthermore, our exploratory analyses examined multiple lifestyle factors simultaneously and increased the likelihood of obtaining significant results by chance.”*

**Point 7:** I noticed that "citizen science" is listed as a keyword, which implies procedural issues, as owners were involved in conducting the experiment. Unlike conventional experiments where factors are tightly controlled, I have concerns about the lack of such control here. Specifically, there was no control over the distance between the box and the cat, the behavior of the cat during Event trials, or pseudorandomization of the target box’s left-right position. These uncontrolled factors may introduce too many potential confounds affecting the experiment.

*Response: This reviewer is correct that citizen science may introduce procedural issues (such as the distance between the boxes as mentioned in Point 9 (originally: Minor Point 2). Owners were verbally instructed on how to move the boxes in real-time by the researcher during the trials, but this still allowed room for minor procedural errors. We have added a sentence to clarify that owners were verbally instructed what to do during the trials as this information was previously missing.*

*Lines 204 – 206 “Before the trials, the researcher physically demonstrated to the owner how they should carry out the study in relation to moving the boxes, the toy and the folder. The researcher also verbally instructed the owner what to do during the trials.”.*

*It is also true that citizen science experiments do not have as tight controls over extraneous variables, which in our study would include variables such as the starting distance of the cat. This was implemented with the hope this would have encouraged the cat to engage in experimental trials. A lack of motivation has been a prevailing challenge for cat cognition research (Chijiwa et al., 2021a, 2021b; Pagé & Dumas, 2009), of which citizen science provides a feasible alternative research method which encourages motivation and participation in experimental trials.*

*We have now explained this limitation of citizen science methods in the Discussion section. We hope this provides a fairer representation of both the benefits and limitations of citizen science research.*

*Lines 517 – 523 “Another limitation that should be mentioned is that, by implementing a citizen science design, there was a lack of control over confounding variables such as the starting distance of the cat. This design choice was made to encourage motivation and engagement throughout the trials. Although we attempted to control for some of these variables in statistical analysis (limited by the small sample size), readers should be made aware that confounding variables may have also interfered with our findings. Some confounding variables, such as the left-right position of the hiding box, are capable of being controlled in future studies.”*

*Lines 524 – 530 “This study was designed for administration in cats’ home environments. We had expected that our cat-friendly adaptations would foster recruitment, but this did not turn out to be the case—uptake of our social media recruitment efforts was relatively low. We also expected that cats would be more motivated to participate by allowing flexible starting distances and freedom of movement during trials. However, a substantial minority (42%) of these cats made no apparent effort to search for the toy in the simplest presentation (SVD trials).”*

Minor points

**Point 8:** Lines 20-204 state that low-density foam was adhered inside the boxes to prevent auditory cues. However, cats have more acute hearing than humans. Was it experimentally confirmed that no auditory cues were actually produced?

*Response: The low-density foam was adhered to the boxes to reduce the sound from the toy being placed inside the box. It was not experimentally confirmed that no auditory cues were produced. As this reviewer correctly points out, because cats have more acute hearing than humans, it is not accurate to conclude that sounds cues were not produced at all. The word ‘prevent’ was not an accurate description of the purpose of the low-density foam and instead we have replaced this with ‘reduce’.*

*Lines 180 – 181 “The researchers glued low density foam (1/2” thickness) to the inside of the boxes to help to reduce sound cues from the toy being placed inside.”*

**Point 9:** Around Line 219, two boxes are presented side by side to the cats. What was the distance between the boxes? (Apologies if I missed this detail.)

*Response: In order for the two boxes to be completely hidden behind an A4-sized folder, the two boxes were positioned next to one another. Owners were instructed to position the boxes a couple of centimetres apart, but some owners positioned the boxes together, so they were physically touching. Provided that both boxes were hidden behind the folder, the boxes could either have no physical distance between them, or a very small distance of only a couple of centimetres separating them, but this was not standardised in the current study. This detail was not previously reported in the Methods section but has been made clearer in the resubmitted manuscript.*

*Lines 178 – 180 “The two boxes were instructed to be positioned next to each other and to be separated by a small distance (approx. 2 cm).”*

*In the discussion section, we acknowledge that this is a limitation of our study design because cats may not have perceived the two boxes as two separate hiding locations.*

**Point 10:** Line 230 notes that the bystander was present in the same room during the experiment. What was their approximate distance from the cat? Since this likely varied by room setup, even a range would be helpful.

*Response: We agree with this reviewer that an approximate range for where the bystander was sitting would be helpful for readers to understand the overall setup of the experiment. We have included two sentences in the Methods section to clarify the distance of the bystander.*

*Lines 211 – 213 “Depending on the layout and size of the room, the bystander would sit between 1 m to 3 m away from the apparatus. The bystander and the box manipulator would always swap positions, so the bystander was always in the same place for every trial.”*

**Point 11:** Lines 237 & 248 state that the manipulator "sat neutrally" after the manipulation. What about their gaze and facial orientation?

*Response: Thank you for calling attention to this wording. We have now refined this description of the manipulator to better describe their attentive state during the trials. Because the manipulator was allowed to look toward and talk to the cat to encourage motivation, we have removed the word ‘neutral’ to avoid misinterpretation. We have more accurately labelled the manipulator as attentive instead of neutral when describing the procedure in the Single visible displacement trials and Knowledge-consistent event trials subheadings.*

*Lines 220 – 222 “The manipulating person then sat for 60 s and waited for the trial to end. During this time, the manipulating person was attentive to the cat (i.e. facing and looking towards the cat) and was allowed to speak to the cat to encourage their participation (e.g., “Where’s the toy?”).”*

*Lines 232 – 233 “The manipulator then waited 30 s in an attentive state for the trial to end.”*

**Point 12:** Lines 243-244: As previously mentioned, was the cat allowed to move freely while the manipulator conducted the operation?

*Response: The cat was allowed to move freely while the manipulator carried out the box manipulation. We have now included a sentence to clarify this in the Procedure subheading.*

*Lines 202 – 204 “During the trials, the owner and the researcher were permitted to talk to each other, and the cat was allowed to move freely during the trials.”*

**Point 13:** Line 258: It does not appear to be explicitly stated that the 72 Event trials are divided between the manipulator being either the owner or the researcher.

*Response: We have now clarified that the event trials were divided equally between the owner and the researcher.*

*Lines 244 – 246 “Similar to SVD trials, there were 18 consistent events and 18 violation events with the owner, and there were 18 consistent events and 18 violation events with the researcher.”*

**Point 14:** It was unclear whether left and right are described from the perspective of the cat or otherwise.

*Response: Left and right are described from the perspective of the box manipulator. We agree that it was unclear how this was described in the Methods section and have now clarified this in the ‘Single visible displacement trials’ subheading.*

*Lines 218 – 219 “The cat would see the toy get placed inside either the left or right box (from the perspective of the box manipulator) at random.”*

**Point 15:** Line 507: The authors mention the small sample size as a limitation, but was any effect size or post-hoc power analysis conducted?

*Response: We did not conduct any effect size or post-hoc power analysis for this study. We base this limitation explanation based on the singularity issues that arose in statistical analysis, lending evidence to the global effects of a small sample size in this study. We strove to recruit as many cats as possible in the sampling environment, however, as also mentioned in the manuscript, it is difficult to recruit and retain a large sample of cats for cognitive study.*

Reviewer #2

**Point 16:** This is an interesting study on cat behavior. The manuscript was in good condition. The discussion was adequatley based on the results, and the authors drew conclusions in line with the data,

*Response: We thank the reviewer for these positive assessments of our manuscript.*

**Point 17:** However, the findings were not linked to an overall discuss of how these results improved our understanding of object permanence. Essentially, the final paragraph of the discussion should be on the same rough level as the first paragraph of the introduction.

*Response: We have now amended the final paragraph of the discussion to discuss the findings in relation to our wider understanding of object permanence.*

*Lines 535 – 538 “This warrants further investigation for whether this can be attributed to motivational or cognitive explanations; that is, from the standpoint of object permanence, it is not clear from the present study whether our sample is unrepresentative of cats or whether cats are unrepresentative of mammals in this paradigm.”*

Reviewer #3: Overview and general recommendation:

**Point 18:** This manuscript covers an interesting topic: the object permanence, that is the understanding that objects continue to exist while out of sight. The lack of knowledge and studies in this field on cats requires further research. Thereby, the paper will fit into a gap in knowledge about feline cognition. The article is clearly laid out and all the elements are present, referring to most relevant literature in the area. The authors made a very good job identifying strengths and new insights to be explored with further studies and limitations that need to be considered. For me the paper, a part for a very few subsequent elements, could be accepted in the present form.

*Response: We are grateful for the reviewer’s positive assessment of our submission.*

Specific comments

Abstract
**Point 19:** Please check the length of the abstract: the Plos One guideline gives a maximum limit of 300 words.

*Response: We have now reduced the word count to 300 words. We have also refined the wording of the Abstract for clarity and to accommodate for the reduced word count.*

**Point 20:** For those who are not in the field, it is not clear the paradigm of the permanence of the object compared to the sensorimotor period of a human infant (Piaget, 1954). Perhaps it would be useful to give a very brief explanation of this without taking it for granted.

*Response: Thank you for this suggestion. We have now provided additional context for the paradigm of object permanence within the word limit of the Abstract. We also note that the opening paragraph of the introduction outlines the sensorimotor stages in more detail for additional information that could not be included in the Abstract word limit.*

*Lines 12 – 13 “Object permanence, the understanding that objects continue to exist while out of sight, is a key part of the sensorimotor stage of cognitive development.”*

Introduction

**Point 21:** Please add the title “Introduction” heading at the beginning of the introduction section

*Response: The Introduction heading has now been included at the beginning of the introduction section as per the journal formatting guidelines.*

**Point 22:** Lines 100-125: I would probably move this part in the discussion: it is fine just to mention the reason for your choice, but the argument of discussion seems too broad in this session, and I would prefer to move it into the discussion session

*Response: Thank you for this suggestion; we agree this section would be better explained in the discussion section.*

*We have reworded this section in the introduction to present an overall view of how person familiarity influences feline behaviour in Lines 95 – 100 “Overall, there is mixed evidence of how person familiarity may influence cat behaviour [52, 53, 54, 55, 56, 57, 58], which has important implications in assessing performance in experimental settings and interpreting cat cognition. Otherwise, the individuality of the cat has also been shown to be a significant contributing factor as to how a cat may behave with an unfamiliar person in relation to, for example, approaches, withdrawals, sniffing and distance between the cat and the unfamiliar person [53, 59].”*

*We have still included these details regarding person familiarity and feline behaviour in the discussion section in Lines 462 – 469 “Previous research has found that cats remain longer in proximity with an object that has been handled by their owner in comparison to a stranger [58], whereas other research has found that person familiarity does not influence behaviours such as duration of proximity or physical contact with humans [52]. In Strange Situation Tests [69], there is mixed evidence as to whether cats differentially change their proximity or play behaviours in the presence of strangers compared to the presence of their owner [55, 56]. Otherwise, cats are able to pass A-not-B trials when ostensive cues are provided by a researcher (an unfamiliar person) but not the owner [57].”*

Materials and Methods

**Point 23:** Lines 150-175: are these cats included in the experiment?

*Response: We have now included a sentence to clarify that these cats were not included in the experiment.*

*Lines 154 – 155 “None of the cats from the pilot study were re-visited for participation in the main study.”*

**Point 24:** Lines 187-191: I would move this part in the result section, because you “didn’t decide” these data but they are the random result of the sampling, right?

*Response: We have now moved the information about cat age, sex, neuter status and outdoor accessibility in the Results section, which can be found in Lines 335 – 339.*

*Relatedly, we have also now corrected a typing error which reported an incorrect number of female cats in the sample (in the original manuscript we reported 12 females, but the sample actually contained 10 females). The mean, standard deviation and age range remains the same and is now consistent with the original files that can be found on Mendeley Data (doi: 10.17632/z38t8242st.1).*

Discussion

**Point 25:** Lines 494-499: in your result you find that mixed breed cats were more likely to play with the toy and initiated play with the toy faster than purebred cats. You rightly supposed that temperaments of the purebred cats represented in your sample (Birman and Ragdoll, that are known to be gentle and sociable but not very playful) could be the reason for your result. But it is important underling that you have just 5 purebred cats and this makes the comparison not so effective.

*Response: Thank you for bringing this to our attention. We agree that the small number of purebred cats makes this comparison to the 13 mixed breed cats in our sample less effective. We have now included a sentence in the Discussion section to clarify this shortcoming.*

*Lines 495 – 498 “However, our sample included only five purebred cats in total (three Ragdoll, two Birman), so breed comparisons in toy-directed behaviour are limited by the small number of purebred cats in comparison to mixed breed cats.”*

References

**Point 26:** Please check the format of the reference list; below is reported a part of the guideline for authors:
“Formatting references
Because all references will be linked electronically as much as possible to the papers they cite, proper formatting of references is crucial.
PLOS uses the reference style outlined by the International Committee of Medical Journal Editors (ICMJE), also referred to as the “Vancouver” style. Example formats are listed below. Additional examples are in the ICMJE sample references.
A reference management tool, EndNote, offers a current style file that can assist you with the formatting of your references. If you have problems with any reference management program, please contact the source company's technical support.
Journal name abbreviations should be those found in the National Center for Biotechnology Information (NCBI) databases.
Source Format
Published articles
Hou WR, Hou YL, Wu GF, Song Y, Su XL, Sun B, et al. cDNA, genomic sequence cloning and overexpression of ribosomal protein gene L9 (rpL9) of the giant panda (Ailuropoda melanoleuca). Genet Mol Res. 2011;10: 1576-1588.
Devaraju P, Gulati R, Antony PT, Mithun CB, Negi VS. Susceptibility to SLE in South Indian Tamils may be influenced by genetic selection pressure on TLR2 and TLR9 genes. Mol Immunol. 2014 Nov 22. pii: S0161-5890(14)00313-7. doi: 10.1016/j.molimm.2014.11.005.”

*Response: Thank you for this information and examples regarding reference formatting. The references and in-text citations have now been correctly formatted to follow the Vancouver referencing style instead of APA referencing style.*

**Point 27:** [Missing references]
-Line 59: The reference “(Holekamp, 2007)” is missing in the reference list.
-Line 65: The reference “Dumas (1992)” is missing in the reference list.
- Line 108: The reference “(Vitale & Udell, 2019)” is missing in the reference list.
- Lines 160-161: The reference “(Pyari et al., 2023)” is missing in the reference list.

*Response: Holekamp (2007), Dumas (1992) and Vitale and Udell (2019) are now included in the reference list. Pyari et al. (2023) was attributed the incorrect year, which has now been corrected to Pyari et al. (2022).*

**Point 28:** the subsequent references are missing in the text:
1. Chijiiwa, H., Takagi, S., Arahori, M., Anderson, J. R., Fujita, K., & Kuroshima, H. (2021a). Cats (Felis catus) show no avoidance of people who behave negatively to their owner. Animal Behavior and Cognition, 8(1), 23-35. [https://doi.org/10.26451/abc.08.01.03.2021](https://url.uk.m.mimecastprotect.com/s/GZhTCvZ9NF7W0VylCyCgHQaD9I?domain=doi.org)
2. Chijiiwa, H., Takagi, S., Arahori, M., Hori, Y., Saito, A., Kuroshima, H., & Fujita, K. (2021b). Dogs and cats prioritize human action: choosing a now-empty instead of a still-baited container. Animal Cognition, 24, 65-73. [https://doi.org/10.1007/s10071-020-01416-w](https://url.uk.m.mimecastprotect.com/s/J2amCwrROTGLZrRNCQFzHJ7uM7?domain=doi.org)
3. Koo, T. K., & Li, M. Y. (2016). A guideline of selecting and reporting intraclass correlation coefficients for reliability research. Journal of Chiropractic Medicine, 15(2), 155– 163. [https://doi.org/10.1016/j.jcm.2016.02.012](https://url.uk.m.mimecastprotect.com/s/FXTMCx17PU1JqNQXfAHoHy6MbZ?domain=doi.org)
4. Majecka, K., & Pietraszewski, D. (2018). Where’s the cookie? The ability of monkeys to track object transpositions. Animal Cognition, 21, 603-611. [https://doi.org/10.1007/s10071-018-1195-x](https://url.uk.m.mimecastprotect.com/s/fRo1CyXJ0crNmVy4ukIgHx2vT-?domain=doi.org)
5. McHugh, M. L. (2012). Interrater reliability: the kappa statistic. Biochemia Medica, 22(3), 276-282. [https://doi.org/10.11613/BM.2012.031](https://url.uk.m.mimecastprotect.com/s/F79wCzXxRcMRNVmriJS2H9e5jd?domain=doi.org)
6. Schober, P., Boer, C., & Schwarte, L. A. (2018). Correlation coefficients: appropriate use and interpretation. Anesthesia & Analgesia, 126(5), 1763–1768. [https://doi.org/10.1213/ANE.0000000000002864](https://url.uk.m.mimecastprotect.com/s/VCr_CA19wUN9Jq1RFJTDHGyq4Q?domain=doi.org)

*Response: Thank you for identifying these missing references. We had incorrectly referred to ‘Chijiiwa et al., 2021a, 2021b’ as ‘Chijiwa et al. 2021a, 2021b’ (with a single ‘i’), which has now been corrected with the Vancouver referencing style. We included the other references in an older version of the manuscript which are no longer required for the current version of the manuscript.*

*We have also removed two additional references from the reference list which are no longer required for the current version of the manuscript:*

*Curtis, T. M., Knowles, R. J., & Crowell-Davis, S. L. (2003). Influence of familiarity and relatedness on proximity and allogrooming in domestic cats (Felis catus). American Journal of Veterinary Research, 64(9), 1151-1154.* [*https://doi.org/10.2460/ajvr.2003.64.1151*](https://doi.org/10.2460/ajvr.2003.64.1151)

*Deputte, B. L., Jumelet, E., Gilbert, C., & Titeux, E. (2021). Heads and tails: An analysis of*

*visual signals in cats, Felis catus. Animals, 11(9), 752.* [*https://doi.org/10.3390/ani11092752*](https://doi.org/10.3390/ani11092752)

Figures

**Point 29:** Please add the title in the figures

*Response: The title is now in bold text as per the journal formatting standards. Please also see point 3 in Journal Requirements.*
