# Supplementary material for: Object permanence in domestic cats (Felis catus) using violation-of-expectancy by owner and stranger
Source: PLoS One. 2025 Jul 9;20(7):e0312225. doi: 10.1371/journal.pone.0312225 (PMC12240391; doi:10.1371/journal.pone.0312225)
Supplement: Attachment — Submitted filename: response_to_reviewers_30_05_25.docx [file pone.0312225.s003.docx]

We thank the Editor and reviewers for providing further improvements for this manuscript. We agree that gaze and facial orientation may have exerted an effect on the results and we have now included this in the Discussion section. We also agree that effect sizes should still be reported alongside our analyses to provide more information on the suitability of the sample size. We have responded to the remaining comments below.

Due to saving errors, our track changes were inadvertently removed from the track changes document. We have instead highlighted the track changes in yellow. Apologies for this inconvenience.

**Reviewer Comments**

Reviewer #1: The authors have responded sincerely to most of my questions and comments.
However, I would like to request two further improvements regarding their revisions and responses.

**1.** For my question, “Lines 237 & 248 state that the manipulator ‘sat neutrally’ after the manipulation. What about their gaze and facial orientation?”, the authors addressed it by adding information about the manipulator’s behavior during the trials. However, if their description is accurate, the manipulator’s behavior could have had an effect on the results. This should be acknowledged in the discussion as a limitation.

*Response: Thank you for this further suggestion. We also acknowledge that gaze and facial orientation may have had an effect on the results and have now included this in the discussion as a limitation. Our reasoning for this was to promote the normalcy of the home environment and to encourage participation and interaction with the apparatus. An unfamiliar person in the cat’s home environment, with the additive influence of that stranger behaving in an unexpected way (e.g., sitting still with no discernible facial expression), would likely have further discouraged the cat from participating in the study. We also note that standardised protocols for interacting with cats during cognitive studies are yet to be established, although there is available research that shows that cats are more interactive with attentive humans (in comparison to passive humans). We have now included this information in the Discussion section.*

*Lines 527 – 543 “Another limitation that should be mentioned is that, by implementing a citizen science design, there was a lack of control over confounding variables such as the starting distance of the cat and the exact gaze and facial expression of the box manipulator. These design choices were made to encourage motivation and engagement throughout the trials. Although we attempted to control for some of these confounding variables by including them in the statistical models, readers should be made aware that such variables may have also interfered with our findings. Previous research has found that cats had more frequent approaches and longer durations of time in proximity with attentive humans versus passive humans [59, 52], as well as longer durations of head rubbing and play behaviour with attentive versus passive humans [80], so the attentional display of the box manipulator may have encouraged cats to interact with the toy in some trials more than others, although this would require further investigation. Standardised protocols for interacting with cats in cognitive research are yet to be established, so, due to the cats’ general disposition for avoiding strangers and being easily disturbed by changes in their environment, we chose to maintain as much of the normalcy of the home environment as possible during the trials. However, some confounding variables, such as the left-right position of the hiding box, are capable of being controlled in future studies.”*

**2.** For another question, “Line 507: The authors mention the small sample size as a limitation, but was any effect size or post-hoc power analysis conducted?”, they responded that they did not conduct such analyses. However, effect size and post-hoc power analysis can be performed even with a small sample size. The results of these analyses should be provided for the readers.

*Response:*

***Effect sizes***

*Thank you for this suggestion. We agree that effect size calculations can be performed on linear regression models and have now reported this in the revised manuscript. Please note that odds ratios and incidence rate ratios were already calculated for logistic and Poisson regression models, respectively.*

*For effect sizes, generalised eta squared values were calculated for linear regression models. This is recommended for repeated measures designs (Bakeman, 2005; Cohen, 1988, 2013).*

*The newly-reported effect sizes are as below (which are also reported in the revised manuscript text next to their respective analysis):*

| **Outcome variable** | **Predictor variable** | **Test statistic** |
| --- | --- | --- |
| SVD trials: Gaze duration at box manipulator | Single/dual-cat household | F_1,30_ = 4.44, *p* = .043, ***η*^2^*_G_* = .13, 95% CI 0.00 – 1.00** |
|  | Hiding box | F_1,30_ = 4.43, *p* = .043, ***η*^2^*_G_* = .11, 95% CI 0.00 – 1.00** |
|  | Starting distance | F_3,30_ = 4.55, *p* = .009, ***η*^2^*_G_* = .28, 95% CI 0.04 – 1.00** |
|  |  |  |
| Event trials: Gaze duration at toy | First event | F_1,68_ = 6.16, *p* = .015, ***η*^2^*_G_* = .08, 95% CI 0.01 – 1.00** |
|  | First person trial | F_1,68_ = 5.71, *p* = .019, ***η*^2^*_G_* = .07, 95% CI 0.00 – 1.00** |
|  | Cat sex | F_1,68_ = 6.84, *p* = .010, ***η*^2^*_G_* = .09, 95% CI 0.01 – 1.00** |
|  |  |  |
| Event trials: Latency to play with toy | Outdoor access | F_1,22_ = 5.26, p = .031, **η^2^_G_ = .11, 95% CI 0.00 – 1.00** |
|  | Breed | F_1,22_ = 19.44, p < .001, **η^2^_G_ = .42, 95% CI 0.16 – 1.00** |
|  | Starting distance | F_3,22_ = 4.16, p = .017, **η^2^_G_ = .21, 95% CI 0.00 – 1.00** |

*From the table above, there is a clear trend for wide confidence intervals (spanning from 0.00 – 1.00). This means that there is an increased chance of a Type II error occurring (Lee, 2016) and that we did not have a sufficient number of cats in our sample to have precise estimates. In larger sample sizes, the confidence intervals are typically narrower, and the interpretation is generally more reliable, even if the p value remains constant (Lee, 2016). Although our study found significant differences and small to medium effects between some of the variables (as shown by the p values of < .05 and effect sizes of < .26), the interpretation of the significance level is not reliable (as shown by the wide confidence intervals). This provides statistical support for the limitation of a small sample size, which we had already suspected, and means that some variables with no significant predictors (such as which box the cat checked first) may have a different result if reassessed in future studies.*

*Lines 311– 313 “Effect sizes were calculated using generalized eta squared (η^2^_G_). Effect sizes were interpreted as small (> .02 x < .13), medium (> .13 x < .26), or large (x > .26) [64, 65].”*

*Lines 511 – 517 “Our statistical analyses were limited by our small sample size (as indicated by the wide confidence intervals), and so further research should be carried out using larger samples to further our knowledge on how cats perceive consistent and violation events in the presence of familiar and unfamiliar people. Some variables which had no significant predictors, such as the first box the cat checked, had very wide confidence intervals, which indicate an increased chance of a Type II error [77], so could be reassessed in future studies with larger sample sizes. However, we acknowledge that recruiting and retaining house cats for cognitive studies is challenging.”*

***Post hoc analysis***

*We have reviewed the literature for theoretical perspectives on post hoc power analysis and have also consulted with statisticians at our institution. We do not wish to refute a reviewer comment, however, we also do not want to ignore the vast amount of literature that argues that post hoc power analyses are an unsuitable way to assess statistical power, and is not generally recommended for research (Heckman et al., 2022; Hoenig & Heisey, 2011; Levine & Ensom, 2001). There are some key differences in how population and sample means and standard deviations are calculated between a priori and post hoc power analysis, and because we already know our population parameters, this can have significant consequences on the resultant power statistic (Zhang et al., 2019). A statistical test will always have low observed power when non-significant effects are reported (Goodman & Berlin, 1994), so calculating the post hoc power does not provide any more information when we already know which variables are (non-)significant (Heckman et al., 2022). Furthermore, these differences can be even more exaggerated when using smaller sample sizes (Zhang et al., 2019), which was observed in the current study.*

*Even though the literature strongly suggests that post hoc power analyses should not be used to understand statistical power and sample size suitability, we would like to acknowledge that a priori power analyses are an ideal way to assess power and are important for interpreting results in the context of effect sizes, power, and p values. To strengthen any projects that we may carry out in the future, we will endeavour to have an a priori sample size calculation carried out before any data collection takes place.*

*We are hoping this response is satisfactory for addressing the underlying concern that our models were underpowered due to small sample sizes, and that the reporting of the effect sizes and confidence intervals have provided more context to the interpretation of our results.*

*References:*

*Bakeman, R. (2005). Recommended effect size statistics for repeated measures designs. Behavior Research Methods, 37, 379-384.*

*Cohen, J. (1988). The effect size. Statistical power analysis for the behavioral sciences. Abingdon: Routledge, 77-83.*

*Cohen, J. (2013). Statistical power analysis for the behavioral sciences. Routledge.*

*Goodman, S. N., & Berlin, J. A. (1994). The use of predicted confidence intervals when planning experiments and the misuse of power when interpreting results. Annals of internal medicine, 121(3), 200-206.*

*Heckman, M. G., Davis, J. M., & Crowson, C. S. (2022). Post hoc power calculations: an inappropriate method for interpreting the findings of a research study. The Journal of Rheumatology, 49(8), 867-870.*

*Hoenig, J. M., & Heisey, D. M. (2001). The abuse of power: the pervasive fallacy of power calculations for data analysis. The American Statistician, 55(1), 19-24.*

*Lee, D. K. (2016). Alternatives to P value: confidence interval and effect size. Korean journal of anesthesiology, 69(6), 555.*

*Levine, M., & Ensom, M. H. (2001). Post hoc power analysis: an idea whose time has passed?. Pharmacotherapy: The Journal of Human Pharmacology and Drug Therapy, 21(4), 405-409.*

*Zhang, Y., Hedo, R., Rivera, A., Rull, R., Richardson, S., & Tu, X. M. (2019). Post hoc power analysis: is it an informative and meaningful analysis?. General psychiatry, 32(4), e100069.*
